# Implicit, Predictive Timing Draws upon the Same Scalar Representation of Time as Explicit Timing

**DOI:** 10.1371/journal.pone.0018203

**Published:** 2011-03-25

**Authors:** Federica Piras, Jennifer T. Coull

**Affiliations:** 1 Institute of Medical Psychology and Behavioral Neurobiology, Eberhard-Karls-University Tubingen, Germany; 2 Santa Lucia Foundation, Rome, Italy; 3 Laboratoire de Neurobiologie de la Cognition, CNRS and Université de Provence, Marseille, France; Duke University, United States of America

## Abstract

It is not yet known whether the scalar properties of explicit timing are also displayed by more implicit, predictive forms of timing. We investigated whether performance in both explicit and predictive timing tasks conformed to the two psychophysical properties of scalar timing: the *Psychophysical law* and *Weber's law*. Our explicit temporal generalization task required overt estimation of the duration of an empty interval bounded by visual markers, whereas our temporal expectancy task presented visual stimuli at temporally predictable intervals, which facilitated motor preparation thus speeding target detection. The Psychophysical Law and Weber's Law were modeled, respectively, by (1) the functional dependence between mean subjective time and real time (2) the linearity of the relationship between timing variability and duration. Results showed that performance for predictive, as well as explicit, timing conformed to both psychophysical properties of interval timing. Both tasks showed the same linear relationship between subjective and real time, demonstrating that the same representational mechanism is engaged whether it is transferred into an overt estimate of duration or used to optimise sensorimotor behavior. Moreover, variability increased with increasing duration during both tasks, consistent with a scalar representation of time in both predictive and explicit timing. However, timing variability was greater during predictive timing, at least for durations greater than 200 msec, and ascribable to temporal, rather than non-temporal, mechanisms engaged by the task. These results suggest that although the same internal representation of time was used in both tasks, its external manifestation varied as a function of temporal task goals.

## Introduction

Time processing is central to human behaviour and cognition: it allows us to determine what is happening in our environment and when to respond to events. Time itself is one of the few quantifiable aspects of the world and part of the measuring system used by humans and animals to sequence events, to compare their durations and the intervals between them. Event duration is conceived as the time elapsed since a prior marker (*interval timing*) [1], [2], for example the duration of a sensory stimulus or of an empty interval bounded by sensory markers. In some cases a temporal interval is delimited by the temporal regularity of a repeated event, such as the rate of stimulus presentation or the temporal regularity of a motor act. In these cases, the temporal pattern of events can be used to predict and anticipate the next likely moment of event onset (in the case of empty intervals) or offset (in the case of filled durations), so as to optimize motor or perceptual performance [3], [4]–[6]. While conscious and deliberate temporal processing is required for estimation of duration (to state, for example, whether one CD track lasted longer than another), temporal prediction of future events can often be achieved tacitly [4], [5] without recourse to conscious estimates of duration (for example, starting to sing at the precise moment the next track on a much-loved CD begins). These types of timing, both ubiquitous in real world activities, have sometimes been labelled with terms borrowed from the learning literature, *explicit* and *implicit* timing [3], [7]–[10]. These labels highlight the crucial distinction between processes engaged in tasks for which the goal is to provide an overt estimate of elapsed time (explicit timing) as opposed to tasks in which the goal is non-temporal but can, nevertheless, be facilitated by an (apparently incidental) temporal context (implicit timing). Regularly timed stimulus sequences automatically compel attention, inducing temporal expectancies about future onsets. Moreover, stimulus timing has an immediate and primitive impact on expectancies even when subjects attempt to ignore the time structure of sequences or when anticipatory attending is detrimental to the task goal [4]. The distinction between explicit and implicit timing intersects to some degree with the cognitively controlled versus automatic timing systems defined by Lewis and Miall [11].

The fact that time processing can be engaged automatically by the temporal context of a non-temporal task doesn't preclude the deliberate use of a temporal representation to anticipate events. Indeed, temporal expectations are effectively utilized to improve accuracy and speed in tasks with non-temporal goals [4], [12]. The improvement in performance gained from a predictable, temporally regular task structure has been attributed to optimisation of motor processing [13], [14], but may also be mediated by premotoric stages of processing, such as response selection [15] or sensorimotor association [16], or by even earlier sensory processes [17], [18]. Perceptual judgments are not only quicker but also more accurate when stimuli appear when expected [4], [19], [20] indicating that implicit timing is not exclusively motoric.

Whether explicit and implicit timing share the same representation of time is still a matter of debate. One approach to the question involves describing how the temporal structure of the task affects the neural representation of time intervals. For example, Praamstra and colleagues [10] demonstrated that neural activity during presentation of temporally regular sequences of imperative stimuli (i.e. implicit timing), resembled the neurophysiological correlates of an explicit timing task [21], [22]. The characteristics, but not scalp distribution, of slow brain potentials were the same for explicit and implicit timing showing that the two tasks relied on qualitatively similar timing mechanisms even if implemented in distinct neural substrates. At the behavioural level, several statistical techniques, such as correlational or dimensional analyses [7], [9], [23], [24] and multidimensional scaling [25], have been employed to probe the existence of a common timing mechanism engaged during these distinct forms of timed behaviour. These studies [8], [25], [26] showed no significant correlation between explicit and implicit motor timing tasks suggesting a dissociation in their underlying temporal representations.

An alternative approach involves examining the psychophysical features of the timing process underlying different timed behaviours. In a given series of trials, a timing system should provide both validity and reliability [27], being able to generate values as close as possible to a given target and to keep their variability as low as possible, thus minimizing the dispersion of estimates around the target interval. Animal and human timing display both properties, since mean timing estimates vary linearly and usually accurately with temporal standards (*mean accuracy property*) while the variability of temporal performance increases linearly with standard duration (*scalar property of variance*) [28]–[30].

To provide evidence that a common timing process underlies different forms of timed behaviour, most studies have looked for similarities in the relationship between timing variance and the timed interval [8], [31]. By contrast, very few studies [32] have explored whether task context influences the validity (or accuracy) of temporal estimates. In this paper, we ask whether performance in explicit and implicit timing show conformity to both psychophysical relationships specified by scalar timing: the Psychophysical law [33]–[35] and Weber's law. Moreover, in contrast to previous studies that compared explicit and implicit measures of motor timing [7], [8], [26], [36], we compare explicit and implicit timing in a more *perceptual* context. In motor forms of implicit timing, a motor act (e.g. continuous circle drawing) occurs at regular, predictable moments in time, guided by kinematic principles of the motor system. In perceptual forms of implicit timing, on the other hand, an externally defined sensory event (e.g. onset of a visual target) occurs at predictable moments in time, guided by the constant temporal relationship between cues and targets.

*Explicit* perceptual timing was measured through a classic temporal generalization task, in which sensory inputs were timed in order to determine whether or not they were of equal duration. In this kind of task, performance is scalar for durations from 300/400 msec up to 8 seconds [37]. *Implicit* perceptual timing was studied using a reaction-time paradigm in which temporal expectancies were established by warning cues that indicated when a subsequent visual target was likely to occur. Since the term “implicit timing” could be equally used to describe retrospective timing [38], we henceforth refer to our paradigm as a task of predictive timing, so as to avoid ambiguity. In these predictive timing tasks, RTs to targets appearing after a predictable delay are faster than those to targets appearing at a non-cued delay, demonstrating that subjects are likely to be timing the cue-target interval (*temporal-orienting effect* see [39] for a review). Moreover, previous studies have shown that the coefficient of variation in warned reaction time tasks did not vary as a function of standard duration [9], [40], thus conforming to Weber's law, and that the time between movements in a predictive saccade task demonstrated the scalar property of variance [16]. Taken together these results suggest that predictive timing is based on an internal representation of stimulus time and that scalar timing is recruited in these kinds of tasks. The aim of the current study was to directly compare both the accuracy and variability of predictive and explicit forms of perceptual timing to search for evidence of common underlying representations of time.

## Methods

### Subjects

A total of 27 subjects (12 males and 15 females) mean age of 24.9 yrs (SD 3.0; range: 21–31 yr) participated in the study. They were neurologically healthy students with no history of alcohol or drug abuse or psychiatric disorders. They were informed about the general purpose of the study but naïve about the tasks. All subjects volunteered and gave written consent for this study before being enrolled.

Fifteen (6 males and 9 females) subjects (mean age: 25.3 yrs, SD: 2.7 range: 23–31 yrs) underwent the explicit timing task while the remaining 12 subjects (6 males 6 females mean age: 24.3 yrs SD: 3.5 range: 21–31 yrs) performed the implicit timing task (t test for differences in mean age p = 0.36). From the original group of 15 subjects who performed the explicit timing task, only 10 (5 males, 5 females mean age: 25.8 yrs SD: 3.1, range: 23–31 yrs) showed (among blocks) a mean *d'*>1 (more than 75% of correct responses). Data from the remaining 5 subjects were therefore removed from the analysis.

The study was approved by the Ethical Committee of Santa Lucia Foundation.

### Apparatus

Subjects were seated comfortably in a quiet testing room facing an LC computer screen (1600*1200 resolution 1280*768 pixels, frame rate 60 Hz) and were requested to push mouse buttons to give responses in the explicit timing task or to press on the computer keyboard for the implicit timing task. Stimulus presentation and collection of behavioural responses were controlled by a personal computer using the E-Prime 2.0 software (Psychology Software Tools, Inc., Schneider et al., 2002).

### Task 1: temporal generalization task (explicit timing)

#### Experimental task

**Figure 1:**
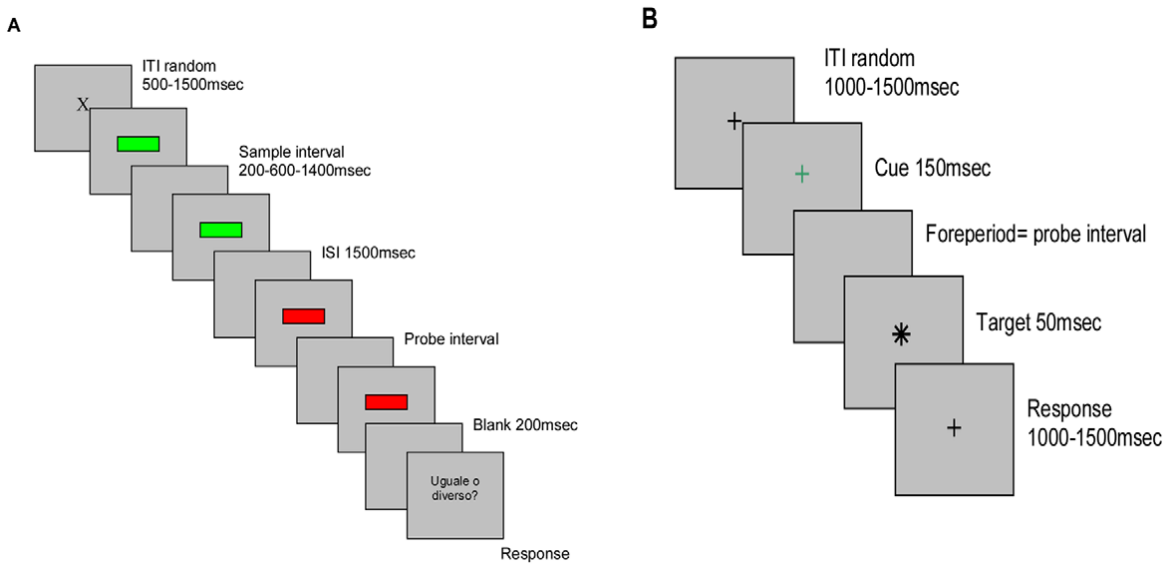
Experimental tasks. **A.** Temporal Generalization (TG) Task. This task measures timing explicitly since subjects are required to provide an overt estimate of interval duration (same/different as trained interval). **B.** Temporal Expectancy (TE) Task. This task measures timing implicitly since subjects are not required to provide an estimate of the cue-target interval (e.g. same/different as expected interval), but instead to make *use* of implicitly acquired timing information to speed reaction time performance. ITI = inter-trial interval, ISI = inter-stimulus interval.

Subjects were trained to distinguish a standard duration (200–600–1400 msec in different blocks) and asked to judge if it was the same or different to a probe (see Figure 1A). Longer durations [3000 and 6200 msec] were also tested in different blocks. Since we did not prevent subvocal counting, which is spontaneously used in temporal tasks for durations above 1500 msec [41], data from long durations were omitted from analyses.

Both the sample and probe intervals were delineated by the elapsed time between two visual signals (onset and offset markers). Subjects had thus, to estimate time-in-passing between two markers and discriminate the second (probe) interval from the first (sample) one. Within a block, the sample interval did not vary across trials (it always corresponded to the standard duration) whereas the probe interval varied on a trial-by-trial basis.

In the training phase (25 trials), the probe interval was always the same as the sample in order to build up a stable reference memory of the standard duration. Participants were required to judge if the probe was equal or different to the sample interval. The experimental task (71 trials each block) was administered immediately after the training phase. Each trial of the experimental task began with a fixation cross (random duration between 500 and 1500 msec), which constituted the inter-trial interval. This was followed by a green bar (103×7 pixels, centered on the screen, duration 100 msec), which represented the onset marker of the sample interval (an empty screen). After the appropriate time had elapsed, a green bar (identical to the previous one) marked the offset of the sample interval. After 1500 msec, the probe interval was presented, whose onset and offset were this time delineated by red bars. A blank screen (duration 200 msec) preceded the prompt for a response (“equal” or “different”?), which remained on screen until the subjects' response.

Subjects were allowed to rest between blocks, and testing lasted approximately 1 hour.

#### Stimuli

Probes were chosen on the basis of previous studies. According to Grondin [42], in the visual modality the ratio of the difference threshold to the standard duration (Weber fraction) is between 12 and 16% of the standard. This fraction is constant for durations ranging from 150 to 900 msec. For each of the standard durations (200–600–1400 msec), probe intervals were varied in eleven steps, centered around the standard duration. Each step corresponded to half of the Weber fraction (6%) [step-sizes: 12 msec for 200; 37 msec for 600; 87 msec for 1.400] so as to achieve as complete a profile of temporal sensitivity as possible. Five probes were shorter than the sample interval (0.7, 0.76, 0.82, 0.88, 0.94 proportion of sample duration), five probes were longer than it (1.06, 1.12, 1.85, 1.25, 1.31 proportion of sample duration) and one probe was of the same length. For example, for the 1400 ms standard interval, we tested 965, 1052, 1139, 1226, 1313, 1400, 1487, 1574, 1661, 1748, 1835 msec probes. Probes within 2 step sizes of the standard and the standard itself were presented 7 times each, while probes 3, 4 and 5 step sizes longer and shorter than the standard were presented 6 times each.

We predicted that probes within 2 step-sizes of the standard would not be perceived as significantly different from the sample (i.e. subjects would produce approximately the same proportion of “equal” responses for these probes as for the standard) while probes at 3, 4 or 5 step-sizes shorter or longer than the standard would be perceived differently from the sample (the proportion of “different” responses would increase with step-size). Accordingly, the conditional probability of intervals within 2 step-sizes of the standard (from 0.88 to 1.12 proportion of the standard, standard included) was set to 50%. Since step-sizes were a constant fraction of the standard duration, difficulty of discrimination was the same for the three standard durations given that the eleven probes were approximately the same proportion of the standard.

#### Variance and CV calculation

**Figure 2:**
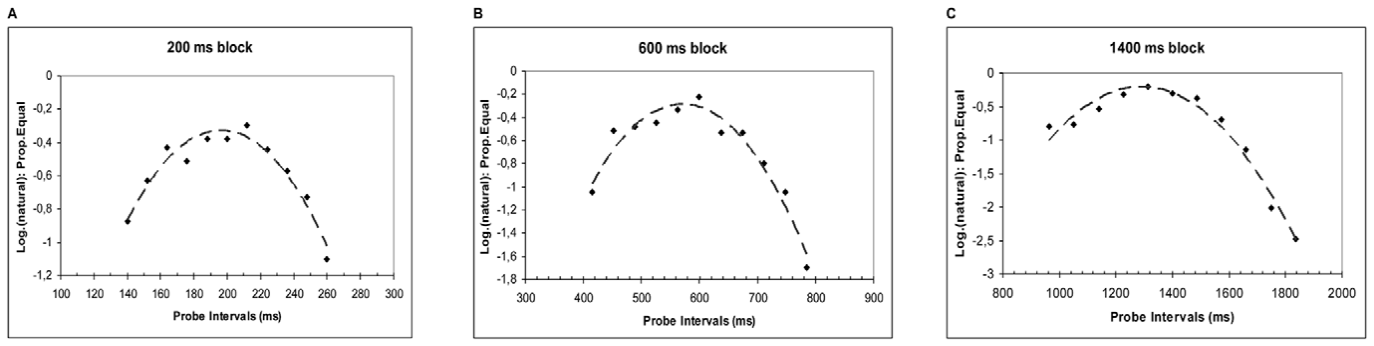
Temporal sensitivity in the explicit timing task. Mean proportion of “equal to sample” responses as a function of the 11 probe intervals for each of the three standard duration blocks (200–600–1400 ms) in the Temporal Generalization task. Polynomial trend lines (dashed) are also shown. For all duration ranges, performance peaks around the standard duration demonstrating accurate timing performance.

The difference threshold, a measure of variability, was computed on the basis of performance from the ten subjects who had a mean *d'*>1. A psychometric function (group data shown in Figure 2) was constructed for each subject by plotting the probability of an “equal to sample” categorization as a function of the 11 probe intervals. The lower and upper thresholds for temporal sensitivity were calculated as the longest/shortest probe intervals where the probability of an “equal to sample” categorization was outside the 95% confidence limit for the mean [43]. The difference threshold, a measure conceptually equivalent to the Just Noticeable Difference (JND), was computed by taking the difference between the upper and the lower thresholds, and dividing this value by two. The coefficient of variation (CV) was calculated as the ratio between the difference threshold and the mean subjective time (average of the upper and lower thresholds, conceptually equivalent to the Point of Subjective Equality, PSE).

Results from the temporal generalization task helped establish the range of probes for the temporal expectancy task. The difference threshold was therefore used as the step-size by which probes would be varied in the temporal expectancy task.

### Task 2: temporal expectancy task (implicit, predictive timing)

#### Experimental task

Subjects were asked to detect, by pressing the keyboard space bar as quickly as they could, the appearance of a target that followed a warning cue by a variable interval (*foreperiod*). In the initial training phase (25 trials) the interval between the warning signal and the target was fixed (200, 600 or 1400 msec in three different blocks), thus allowing subjects to easily establish temporal expectancies about the time of target onset. This training phase was included to induce temporal expectations implicitly: subjects were not instructed to use the intervals they had learnt in the training phase to predict target times in the experimental phase. They were simply asked to respond as quickly as possible. Temporal expectations were further induced implicitly in the experimental phase (171 trials in two consecutive sessions, the second preceded by a new training phase) by differentially weighting the number of trials in which the target appeared after the standard (trained) duration to trials in which it appeared after a non-standard duration. Specifically, the probability that the foreperiod would be the expected (trained) one was set to 60% (*“expected” foreperiod*). In the remaining 40% of trials (*“unexpected” foreperiod*) the foreperiod was drawn randomly from a distribution of 6 shorter and 6 longer intervals (described in the next section). In 10% of total trials the target was not presented (*null* or *catch trials*). This manipulation was introduced to induce “*dispreparation*” [44], [45], which would help attenuate the effects of the “hazard function” (the increasing preparedness invoked by longer foreperiods once the target fails to appear at the expected foreperiod). A sketch of the task is shown in Figure 1B. A fixation cross (centered on the screen, random duration between 1000 and 1500 msec) was followed by a brief visual cue (the fixation cross turned green for 150 msec). After a variable foreperiod, the target was briefly presented (an X superimposed upon the fixation cross, duration: 50 msec) and participants were required to press the spacebar as soon as the target appeared. A new trial started between 1000 and 1500 msec afterwards.

E-Prime software can provide millisecond precision by setting timing from stimulus onset to stimulus offset as the specified duration (timing mode: event). The timing for events other than the cue, the cue-target foreperiod, and the target, was set so that any potential delay in stimulus delivery would accumulate *across events* and be recovered during the inter-stimulus or fixation intervals (timing mode: cumulative).

#### Stimuli

Timing variance from the 10 subjects who, in Experiment 1, showed a mean accuracy above 75%, was used to set the length of the unexpected foreperiods for the TE task and were calculated as: [expected foreperiod ± 3; 2.5; 2; 1.5; 1 or 0.5 of the difference threshold]. The smallest detectable difference for each standard duration, as calculated in Experiment 1, was therefore used as the step-size by which probes would be varied in the present task. We thus derived 6 shorter and 6 longer “probe” intervals. Given that temporal variance in Experiment 1 increased as a function of the standard duration (Weber's Law), the ratio between the probe and the expected foreperiod was slightly different for different standard durations, varying on average no more than 10% (for example, the ratio of shortest probe to the expected one ranged from 0.45 at 200 msec to 0.52 at 1400 msec). In line with the “expectancy profile” described by Jones and colleagues [4], [5] for rhythmically expected stimuli, we predicted RTs to be fastest for the expected foreperiod and significantly slower as a function of how much shorter or longer the unexpected foreperiod was than the expected one (within |1.5| and |3| difference thresholds from the expected foreperiod). On the contrary, intervals within |0.5| and |1| difference thresholds from the expected foreperiod were hypothesized not to show any effect on RTs since the subjects would not be able to discriminate them from the expected foreperiod: the difference threshold, as defined in Experiment 1, identified the smallest detectable difference between two durations. Therefore, all probe durations falling below this threshold would not be perceptibly different from the standard foreperiod.

#### Variance and CV calculation

**Figure 3:**
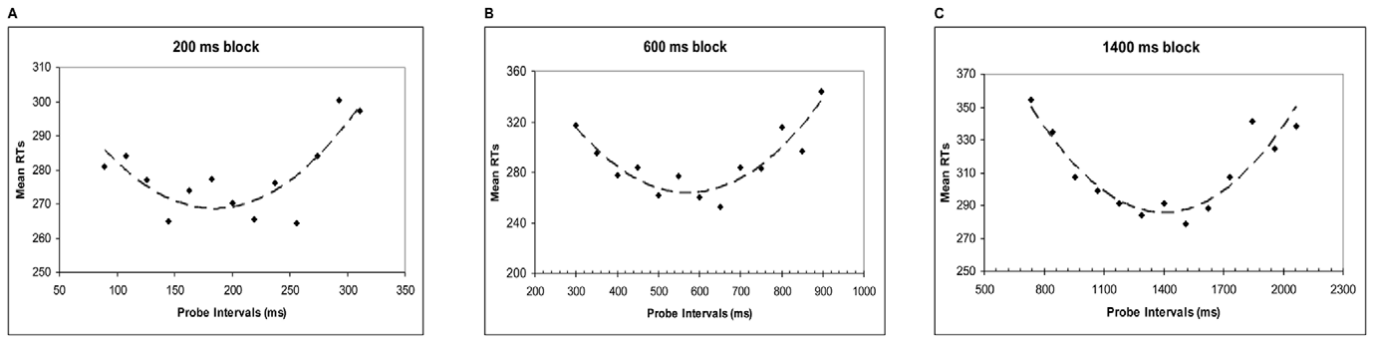
Temporal sensitivity in the implicit, predictive timing task. Mean RTs as a function of the 13 probe intervals for each of the three standard durations (200–600–1400 ms) in the Temporal Expectancy task. Polynomial trend lines (dashed) are also shown. In general, performance peaks around the expected interval, demonstrating accurate timing performance.

A psychometric function (group data shown in Figure 3) was constructed for each subject plotting mean RTs (outliers <100 >1000 msec removed, 0.17 of total responses) as a function of the 13 possible foreperiods. We adopted a U shaped psychometric curve, well suited to capture the effect of temporal expectation, with slowest RTs for the extremely short/long unexpected foreperiods and faster RTs for foreperiods approaching the expected one. Similar to Experiment 1, performance variability was computed as a difference threshold i.e. half the difference between the longest and shortest intervals yielding RTs greater than the 95% upper confidence limits for mean RTs in each block. The mean subjective time was calculated as the average of the two intervals. CV was calculated as the ratio between performance variability and the mean subjective time. Since data from the temporal generalization (TG) and the temporal expectancy (TE) tasks were both well-fitted by U-shaped curves, the same method could be used to define scaled indices of temporal performance (i.e. CE and CV) for each task. These scaled measures were then compared between tasks.

### Analysis of behavioral data

#### Psychometric fitting

For each of the three standard durations (200–600–1400 msec) in the Temporal Generalisation task, three separate second order polynomial regressions were performed on the group average of the logarithm of the mean proportion of equal responses (*Prop.Equal*) across the 11 probe intervals (*t*) (Figure 2). For each standard duration in the Temporal Expectancy task, three separate polynomial regressions were performed between group mean reaction times (*RTs*) across the 13 probe foreperiods (*t*) (Figure 3).

The regression formula for the Temporal Generalisation task was:
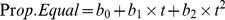
(1)


And for the Temporal Expectancy task was:
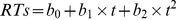
(2)


#### Temporal accuracy

To evaluate if participants had an accurate representation of the standard durations in both tasks we calculated, for each subject, the constant error (CE) as the difference between the mean subjective time and either the sample interval (Temporal Generalization task) or the expected foreperiod (Temporal Expectancy task).

In addition to the CE analysis and to further explore the psychophysical relationship between subjective time (i.e. the perceived interval) and physical time (the standard duration), a power function was fitted to individual data for all standard durations in both tasks:
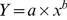
(3)where *a* is the intercept of the regression between mean subjective time and real time, and *b* is the exponent (equivalent to the slope of the linear regression) representing how the psychological magnitude (mean subjective time, which is equivalent to the Point of Subjective Equality, PSE) grows as a function of physical magnitude. The intercept of a power function captures constant processing errors (such as delays in registering the presentation of the stimuli or in reacting to them), while the exponent represents variable processing errors in the timing of a given interval. For the field of time perception the exponent is reported to be close to 1.0 [35], [46] meaning that subjective time *is* real time [47], [48] and that sensitivity to time is constant across different durations. Exponent values from different temporal tasks can be compared to examine whether temporal sensitivity is influenced by the temporal task context.

#### Temporal variability

Another key property of timing behaviour is the variability of time estimates over a series of trials [49]. In psychophysics this relationship is known as *Weber's law* and states that, for any stimulus property (brightness, length etc.), the difference threshold (i.e. the minimum difference between two stimuli that is required to distinguish them) increases monotonically as a function of the magnitude of the standard stimulus. Specifically, the ratio between the difference threshold and the standard duration, namely the *Weber fraction* (WF) or the *coefficient of variation* (CV) is invariant over the magnitude of the stimuli being discriminated [28]. Variance and CV calculation for both tasks is described in the experimental task section.

In addition to the variance analysis and in order to dissociate the time-dependent and the time-independent sources of variation in both tasks, the *slope method* was used. This method [50], [51] presumes that the total variability in a temporal task can be dissociated into a time-dependent component, assumed to reflect central clock-like processes, and a time-independent component thought to reflect variability due to additional psychological operations other than timing mechanisms. The slope method uses a generalized form of Weber's law [52] in which a linear regression between the variability and the squared interval duration is performed. The slope from the linear regression represents variability due to timing processes, while the intercept represents variability due to non-timing processes. Thus, slope analysis provides a method for evaluating whether a similar scalar timing process is being recruited by different temporal tasks, and for comparing temporal acuity in different tasks once the time-independent source of variability has been factored out [51]. It also provides evidence of whether a timed behaviour is influenced by other factors (such as sensorimotor transmission or attentive processes) in addition to actual duration [24]. A linear regression between timing variance (σ^2^) (i.e. the difference threshold) and the standard duration (D^2^) squared was thus, performed.
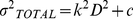
(4)where k (slope) is the constant of proportionality representing the rate of increase of time-dependent variability, D is the duration of the standard (or expected) interval and c (intercept) is the constant representing the time-independent variability component.

## Results

### Psychometric performance

The polynomial fit of group data across the range of 11 (TG task) or 13 (TE task) probe intervals was significant for both tasks. The adjusted R^2^ values were always high (Table 1) and the chosen model accounted for more than 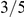 of the total variance. Thus, both data from both explicit (TG task) and predictive (TE task) timing tasks followed a U-shaped curve and could therefore be considered to represent sensitive indices of temporal acuity.

**Table 1:** Polynomial regression values.

Temporal Generalization			Temporal Expectancy	
Duration	Intercept	Ra^2^	Intercept	Ra^2^
**200**	−6.92	91.73	332.67	60.62
**600**	−9.53	92.78	490.14	78.43
**1400**	−12.99	95.90	570.47	81.48

Intercepts and Ra^2^ values from the polynomial regressions fitted to mean (group) scores at each of the 11 (TG task) or 13 (TE task) probe intervals, for each of the three standard durations in either task. See also Figures 2 and 3.

### Temporal Accuracy (mean accuracy property)

To explore if subjects had accurate representations of the standard duration, a mixed ANOVA on CE (Table 3) from both tasks (task [TG and TE] as the between subjects factor and standard duration [200–600–1400 msec] as a within subjects factor) was conducted. The analysis revealed no main effect of task [F_1,9_ = 2.99 p = .11, effect size: 0.47], no effect of standard duration [F_2,18_ = 1.75 p = .20, effect size: 0.58] nor interaction between task and duration [F_2,18_ = 2.32 p = .12, effect size: 1.4]. Thus, subjects were able to form an accurate representation of the standard durations either when they were explicitly discriminated from a probe duration, or when inferred implicitly from foreperiod expectancy effects on RTs.

**Figure 4:**
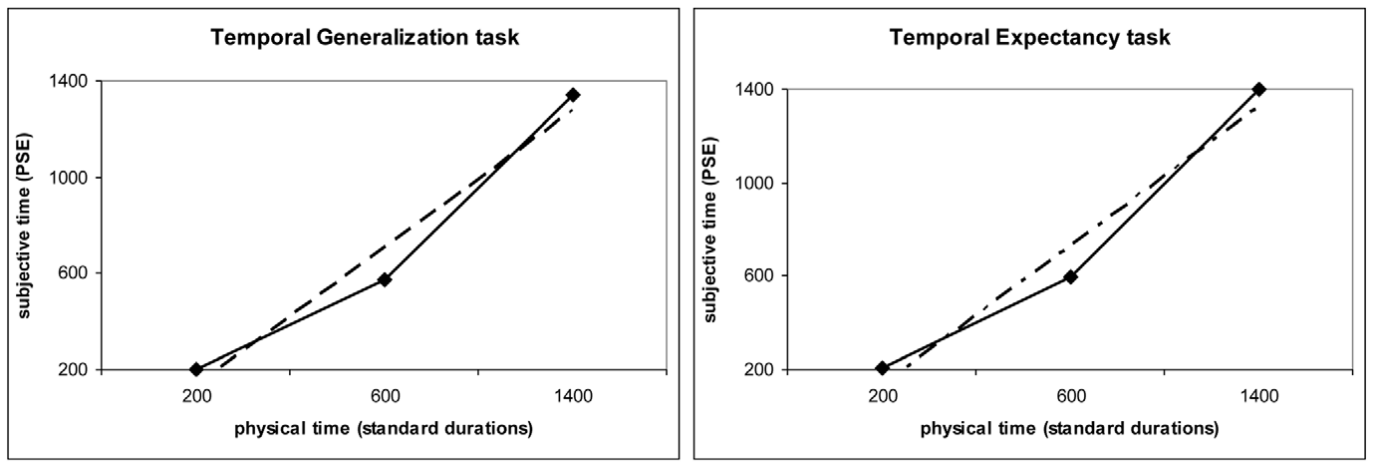
Mean subjective time increases in line with physical time for both explicit and implicit timing. Subjects' mean subjective time (PSE) is plotted as a function of physical time (the standard duration) for the Temporal Generalisation (explicit timing) and Temporal Expectancy (implicit, predictive timing) tasks. Trend lines (dashed) for power functions are also shown.

To further explore whether the task context modulated subjects' time processing sensitivity and consistency, a power function was fitted to individual data for all standard durations in both tasks (Table 2). The exponent values (close to 1) confirm that subjective time varied linearly with real time [46] in both the TG and TE tasks (Figure 4). Moreover, to substantiate our finding, and to confirm linearity, data from a fourth duration (3000 ms) in the TG task were added to the analysis (after having checked *a posteriori* that the coefficient of variation -CV- at that duration did not differ from CVs at shorter durations). In this new regression analysis, the exponent still fell between 0.93 and 1.02, thus confirming that data conformed to a monotonic Psychophysical law in the range of 200 to 3000 ms.

**Table 2:** Parameters (mean ± sd) of the power function fitted to individual data.

Task	intercept (ln)	exponent	R^2^	SE
**Temporal Generalisation**	1.18±0.21	0.97±0.02	0.99±0.002	0.05±0.04
**Temporal Expectancy**	1.16±0.49	0.99±0.07	0.99±0.005	0.06±0.05

These data describe the relationship between mean subjective time and real time (see also Figure 4). The intercept represents non-temporal error and the exponent represents temporal error. Standard error of estimate (SE) is also reported.

A between-subjects ANOVA (with task as the between subjects factor) was conducted on intercept and exponent of the power functions. The task context did not affect the constant processing error due to non-temporal mechanisms (intercept) [F_1,9_ = 0.006 p = .93] nor the subjects' temporal processing sensitivity (exponent) [F_1,9_ = 0.97 p = .34]. Overall these results suggest that the proportionate timing error due to mechanisms other than the clock, and the relationship between psychological time and real time, was the same in both tasks.

### Temporal variability (scalar property of variance)

A mixed ANOVA on variance (i.e. difference threshold, Table 3) from both tasks showed main effects of task [F_1,9_ = 150.60 p<.001] and duration [F_2,18_ = 677.07 p<.001]. A significant task x duration interaction [F_2,18_ = 68.74 p<.001] revealed that variance was the same across tasks at 200 msec (p = .056) but was always significantly higher in the TE task at 600 and 1400 msec (p<.001). Overall, these results indicate that although variance increased as a function of duration in both tasks, the pattern of increase varied as a function of task.

**Table 3:** Psychophysical measures.

	Temporal Generalization				Temporal Expectancy			
Duration	Var (sd)	PSE (sd)	CV (sd)	CE (sd)	Var (sd)	PSE (sd)	CV (sd)	CE (sd)
**200**	44.4 (11.73)	202.4 (9.87)	0.22 (0.05)	2.24 (9.87)	80.55 (16.93)	204.85 (20.7)	0.39 (0.1)	4.85 (20.7)
**600**	142.45 (21.45)	572.25 (36.22)	0.23 (0.03)	−27.75 (36.22)	229.55 (49.35)	594.55 (67.2)	0.39 (0.1)	−5.45 (67.21)
**1400**	343.65 (32.09)	1343.45 (58.18)	0.24 (0.02)	−56.55 (58.18)	566.1 (43.77)	1400 (78.5)	0.40 (0.02)	0 (78.48)

Mean difference threshold (or variance, Var), subjective time (PSE), coefficient of variation (CV) (calculated as difference threshold/PSE) and constant error (CE) (calculated as PSE – standard duration) at each of the three standard durations (200–600–1400 ms) for either the explicit (TG) or implicit (TE) task.

**Figure 5:**
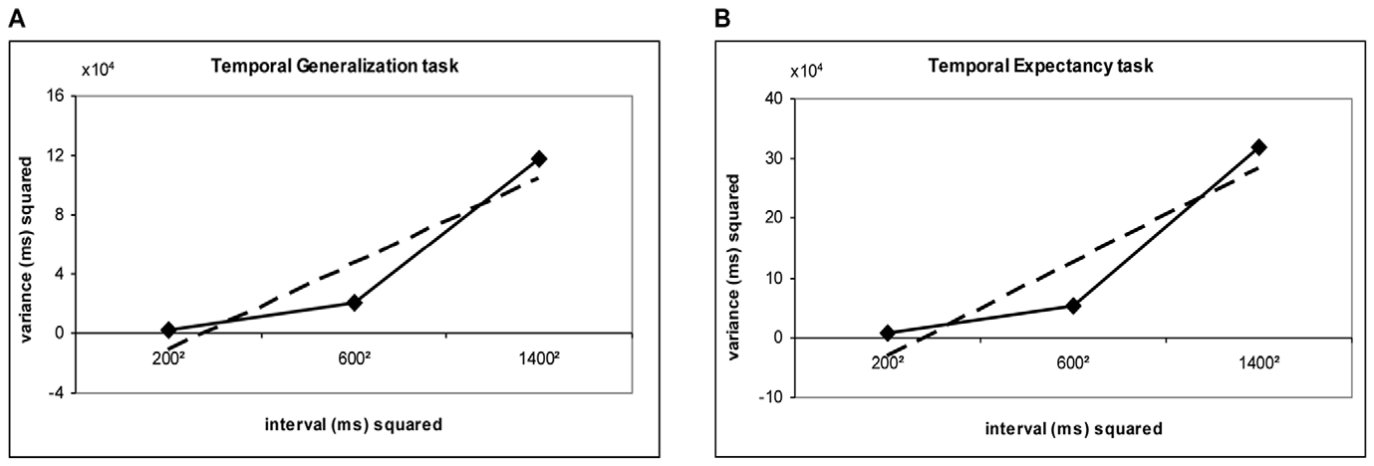
Mean variance increases as a function of duration for both explicit and implicit timing. Subjects' mean variance squared is plotted as a function of the standard duration squared for the Temporal Generalisation (explicit timing) and Temporal Expectancy (implicit, predictive timing) tasks. Trend lines (dashed) for linear functions are also shown.

Given the reported significant interaction, two ANOVAs were conducted on variance separately for the two tasks. A repeated measure ANOVA on variance from the TG task revealed a main effect of duration [F_2,18_ = 455.69 p<.001]. Post-hoc comparisons (Tukey's Honest Significant Difference) showed that variance increased as a function of duration (p = .0001) (Figure 5A). The same ANOVA on variance from the TE task showed again, a main effect of duration [F_2,18_ = 457.4 p<.001]. Post-hoc comparisons (Tukey's HSD) revealed that variance increased as a function of duration (p = .0001) (Figure 5B). Thus, in both tasks, the scalar property of variance was confirmed.

To evaluate if the CV (variance/mean subjective time, Table 3) was invariant over the range of the standard durations considered, as Weber's discrimination law would predict, a mixed ANOVA was performed on CVs from both tasks. The standard duration did not have any effect on CV in the TG task nor in the TE task (no significant effect of duration [F_2,18_ = 0.40 p = .67, effect size: 0.26] nor interaction between task and duration [F_2,18_ = 0.72 p = .49]). Thus, the scalar property was again, confirmed in both TG and TE tasks. However, a main effect of task [F_1,9_ = 77.89 p<.001] was observed. Processing efficiency, as expressed by a lower CV, was better in the TG, compared to TE, task.

Slope analysis was used to further explore temporal variance in the two tasks. Using the generalized form of Weber's law, which is the most accepted expression of the relationship between variance (difference threshold) and duration (real time) [49], a linear regression was performed. Table 4 shows the intercept (c), slope (k^2^) and R^2^ from the slope analysis in both tasks. It is evident that the reported values differed across tasks, although the percentage of variance accounted for by the linear fit (R^2^) was high for both tasks. As before, to further verify linearity, data from the 3000 msec block of the TG task were added to an additional, separate analysis. This 4-point data-set were best fit by a linear, rather than quadratic, regression.

**Table 4:** Parameters of the slope analysis fitted to individual data.

Task	c (squared) (± SD)	slope (± SE)	R^2^(± SD)
**Temporal Generalisation**	−772.19±3605.4	0.06±0.002	0.98±0.0009
**Temporal Expectancy**	−2007.4±12810.06	0.16±0.01	0.98±0.0005

These data describe the relationship between timing variability and the standard duration (see also Figure 5). The intercept (c) represents non-temporal sources of variability and the slope represents timing variability.

Between-subjects ANOVAs were conducted on slope and intercept values. Slope, which represents the time-dependent component of the total temporal variance [51], showed a main effect of task [F_1,9_ = 130.26 p<.001] such that the time-dependent source of variation was higher in the TE task. Task did not affect the time-independent component of the model [F_1,9_ = 0.11 p = .74] with negative intercepts in both tasks. The behavioural context thus modulated only the time-dependent component of temporal processing.

Despite the good fit in the linear regression between timing variance and the interval duration squared, the intercepts showed negative values in both tasks (TG: -9.59 msec [SD 52.27 msec]; TE: -17.65 msec [SD 108.42 msec]). Since the intercept represents duration-independent sources of variance [51], it should be no less than zero. However, t-tests on the intercept term were non significant (p>.05) in both tasks confirming that this variable was not significantly different from 0. Thus, the non-temporal source of variance is negligible in both tasks. Given the reported finding and in order to verify linearity of the TE data-set (in the absence of a fourth duration) a linear function was fitted to individual data with the intercept fixed at 0, thus increasing the total degree of freedoms in the model. In this additional analysis the fit was still significant and slope values did not differ from those determined when the intercept and the slope were free parameters (p values for the t-test>0.05).

## Discussion

In this study we explored the psychophysical (scalar) properties of interval timing for two temporal tasks that differed in the nature of their task instructions. While an overt estimate of the duration of an empty interval was required in the *temporal generalization task*, temporal regularities in stimulus presentation allowed for enhanced preparation and speeded motor performance in the *temporal expectancy task*. These tasks index perceptual forms of explicit and implicit timing [3] respectively, in which duration estimation is overt in one case (*explicit timing*) but incidental, though nevertheless useful for the task goal, in the other (*implicit timing*). Moreover, in our experiment, temporal representations could be acquired implicitly in the temporal expectancy task, without any explicit instruction to encode time (see also [53]). Thus, the terms implicit and explicit refer to task demands and not to the underlying timing processes.

### Both explicit and implicit, predicitve timing demonstrate the scalar property

We asked whether the implicit use of timing to predict stimulus onset and therefore speed up the detection of temporally regular sequences of imperative stimuli, entailed an internal representation of time and, if so, whether this representation had the same psychophysical properties as that required for explicit timing tasks. We therefore examined whether principles of interval timing theory (the *mean accuracy* property and the s*calar property of variance*) were equally valid in both explicit and implicit, predictive timing. Two different mathematical models were applied to our data: a power law functional dependence between mean subjective time and real time, and a linear relationship between timing variability and standard duration. These models allowed us to test whether temporal task goals (explicit/implicit) influenced the internal representation of elapsed time and the proportional error in estimating a given duration.

Previous findings have demonstrated that the relationship between timing variance and timed duration is different in different temporal tasks [7], [8], [26], [36] and is influenced by interval structure (auditory/visual, filled/empty) [54], [55] and sensorimotor context (perception/production) [24], [25]. When timing variability has previously been compared in explicit and implicit measures of *motor* timing, temporal variance differed across tasks, suggesting that separable timing mechanisms were recruited [7], [8], [26], [36]. As yet, no previous study has directly compared variability or accuracy in explicit and implicit forms of *perceptual* timing, in which the temporal properties of sensory stimuli are either explicitly timed or are acquired implicitly to establish temporal expectancies. fMRI studies of temporal expectation have demonstrated that a particular set of regions, most consistently left inferior parietal cortex [56] but also left premotor cortex and cerebellum, are activated when subjects use temporal predictability [10], [57]–[59] or informative temporal pre-cues [60]–[62] to anticipate events. This contrasts clearly with activation of more mid-line structures such as basal ganglia and Supplementary Motor Area, or right-lateralized frontal and temporal cortices, by explicit timing [3], [61], [63] demonstrating that at least the neural substrates of explicit and implicit, predictive timing diverge.

In the current study, purely behavioural comparisons of accuracy and variability in estimating a standard time interval were made across explicit and implicit, predictive timing tasks. Our first key finding is that performance conformed to scalar properties of timing in both tasks. The constant error in estimating the standard duration was negligible, whether estimates were indexed explicitly by perceptual discrimination or implicitly by speeded reactions. Moreover, variability in subjective time increased with mean subjective time in both tasks, demonstrating that performance was scalar for both explicit and implicit timing.

What could these findings indicate? First of all, accuracy data strongly suggest that even if time estimation was not mandatory, but rather incidental to the temporal task goal, elapsed time between the warning signal and the target was correctly estimated during implicit, predictive timing. Reaction times were fastest for targets appearing after the entrained delay and RT variability increased as a function of the trained standard duration. This indicates that a scalar internal mechanism is engaged in implicit, predictive timing, and that the ability to react to temporally predictable sequences of events is under the operation of interval timing. Secondly, when a power function was fitted to individual data, we found that subjective time was linear to real time in both tasks and that subjects' temporal processing sensitivity and consistency was similar for both explicit and implicit timing. This substantiates the hypothesis that the two processes share the same representational mechanism responsible for transforming objective time into psychological time.

The question arises as to which mechanism might underlie the ability to form an accurate interval representation, either when it is transferred into an overt estimate of duration or used to optimise sensorimotor behavior. In the psychological timing literature, by far the most influential model has been the *internal clock model* [64], [65]. In this theoretical account [29], [66], the scalar mechanism responsible for transforming objective time into subjective time is thought to take place at a clock stage, where pulses emitted by an internal pacemaker are transferred via a switch into an accumulator whose content grows as a linear function of real time. As for the neural mechanism underlying timing, that is *“…the neural properties that are actually sensitive to time rather than involved into the readout*” [67], several modelling approaches (*climbing firing rates models*) [68]–[70] propose that a particular pattern of neural activity, observed during tasks involving fixed temporal intervals, may carry duration information. For example, electroencephalographic (EEG) studies have described sensitivity to temporal information in the *contingent negative variation* (CNV), a slow negative wave developing between a warning signal and a subsequent imperative stimulus. Although CNV peak amplitude is constant across different durations, its time-course (onset time, rate of activity increase and peak latency) is duration-dependent, thereby reflecting temporal task properties. The CNV develops during explicit motor and perceptual timing tasks [21], [22], [71]–[73] and also when subjects implicitly adjust their performance to the temporal structure of the task [10]. Indeed, the effect of explicit and implicit perceptual timing on climbing neuronal activity is very similar (see [40], [74] in the animal literature, and [10] for human timing) since activity stops increasing at the end of a memorized duration (when a current test duration has to be compared to a previously learned interval) or at the expected time of stimulus occurrence relative to a preceding cue [69]. Thus, climbing activity might represent the duration of a learned or experienced temporal interval and the physiological mechanism discussed above could be considered the common representational mechanism shared by explicit and implicit, predictive timing.

### Variability in explicit and implicit, predictive timing differs as a function of duration

The second key finding in this study is that although variance increased as a function of duration in both tasks, indicating scalarity, it did so at different rates. Specifically, variance was the same across tasks at 200 msec, but was significantly higher in the implicit task at 600 and 1400 msec. This finding suggests that the same timing process is used for both explicit and implicit, predictive timing at 200 msec, while different processes come into play when estimating longer durations. Indeed, several authors [67], [75]–[80] have hypothesized that intervals in the millisecond and the multisecond range are measured by distinct brain mechanisms. In this regard our results would suggest that for very brief durations (200 msec), time is not estimated explicitly but instead a more automatic mechanism is recruited, which is the same as that engaged in the implicit timing task. Indeed, recent theoretical and experimental work suggests that within the milliseconds range, timing does not rely on clock-like mechanisms or a linear metric of time, but rather is inherently encoded in the state of cortical sensory networks (*state dependent networks models)* [81]–[83]. In other words, the way a neural network evolves through time can itself code for time as a result of time-dependent changes in synaptic and cellular properties. In the framework described above, timing on a brief scale (below 500 msec) [84] would be local, with different parts of the brain being recruited as a function of the somatosensory, auditory, visual or motor task. Speculatively, we can hypothesize that since the explicit and implicit timing measured in our study rely upon visual processing, the shared mechanism for timing a very brief duration (200 msec) may be rooted in visual cortical areas. Indeed, recent studies have shown that the temporal predictability of expected visual events is encoded in a wide network that includes neuronal populations at the very earliest of cortical stages of visual processing [85].

So far we have suggested that the same mechanism is equally engaged in explicit and implicit perceptual timing when subjects estimated the shortest duration. However, the two processes diverge for longer durations. When variability in temporal estimates was modeled, the rate of increase in variability (the slope in the linear regression between timing variance and the standard duration) was larger in the implicit task and ascribable to the temporal, rather than non-temporal, mechanism engaged. Since evidence for a common timing mechanism using slope analysis [50]–[52] rests on accepting the null hypothesis that slopes are equal, we must conclude that perceptual forms of explicit and implicit, predictive timing recruit distinct temporal mechanisms at these longer durations. Therefore, while subjective time was linear to real time in both tasks, mean estimates were more variable, and processing efficiency reduced, in the implicit task. Overall these results demonstrate that whereas the two tasks share a common *representation* of elapsing time, putatively embedded in climbing neuronal activity, distinct task requirements (at least at durations of 600 ms or more) might recruit distinct timing *mechanisms*, which induce different degrees of temporal variability.

It is possible that representations of duration, encoded in the brain in a particular pattern of neural activity, can be rooted in distinct context-specific brain areas, thus accounting for divergence in different timing behaviours. Indeed, even though fMRI data have shown that some key regions, such as the basal ganglia, the supplementary motor area and the prefrontal cortex, are consistently activated during different explicit timing tasks [3], [63], [86], [87] various other cortical regions provide more context-dependent representations of duration [78], [88]–[90]. More specifically, anticipatory neural activity has been observed in a variety of task-specific processing areas during implicit timing, including lateral premotor cortex [10] motor cortex [40], parietal cortex [91] and visual cortex [17]. Differences in temporal performance, and particularly in the variability of time estimates, may therefore be accounted for by context-induced changes in the level of participation of specific neural structures to the distributed timing network [24].

Temporal variability may have also been influenced by behavioural elements present in the timing paradigms. In fact, even if cognitive factors (such as attentive processes or sensorimotor transmission) were equated in the two tasks, as substantiated by the observation that the non-temporal source of variance was negligible in both tasks, our tasks differed in two crucial aspects that might have influenced time-dependent sources of variability. First, probes were more closely spaced around the standard in the temporal generalization task, and a narrower spacing of probes (i.e. a more difficult discrimination) has been proven to affect the decision stage of the timing process with a lower (i.e. more “strict”) threshold in the most difficult discrimination [92], [93]. Second, repeated presentation of the standard (presented on each trial in the temporal generalization task only) could have reduced temporal variability, leading to a sharpened representation and a more efficient encoding of the target duration in reference memory [51].

### Conclusions

Our data demonstrate that the scalar property of timing holds for implicit, as well as explicit, forms of perceptual timing. However, by separately modelling accuracy and variability of time estimates, we reveal a functional dissociation in these two forms of timing. While accuracy of estimating elapsed time is equivalent for explicit and implicit tasks, the increase in variability of temporal estimates as a function of time is greater during implicit, predictive timing. We propose that while the same neural property, the linear ramping of neuronal activity over time, may code for elapsed time in both tasks (at least for durations of 600 ms or more), temporal task goals (explicit/implicit) determine to what extent specific timing mechanisms, and therefore distinct brain regions, participate in the distributed system for interval timing and, thus, modulate subjects' performance. Future experiments will employ a greater number of standard durations in order to confirm the linearity of our results, and will help define more precisely the point at which these two forms of timing begin to diverge.
